# Book Reviews

**Published:** 1891-06

**Authors:** 


Essentials of Surgery, together with a Full Description of the Handkerchief and Roller Bandage. Arranged in the form of questions and answers; prepared especially for students of medicine. By Edward Martin, A. M., M. D., Instructor in Operative Surgery, University of Pennsylvania; Surgeon to the Howard Hospital; Assistant Surgeon to the University Hospital. Sanders' Question Compends No. 2. Illustrated; fourth edition, revised and enlarged by an appendix, containing full directions and prescriptions for the preparation of the various materials used in Antiseptic Surgery. Also several hundred receipts covering the medical treatment of surgical affections. Philadelphia: W. B. Sanders. 1891. Price, $1.00.

This volume is one of the best of the Question-Compends, having passed through three previous editions. It is no longer a stranger to the student, the quiz-master, or the reviewer. When we saw the first edition, two years ago, we regarded it with as much favor as we can bestow on any question book. It is impossible to acquire a knowledge of surgery within the lines covered by such a work; nevertheless, it may serve to refresh one's memory, or to clear up an intricate point, without searching through a general treatise. The illustrations are fairly good, while the letter-press, paper, and make-up of the volume are excellent. It is more comprehensive than such books are in general, and we com mend it to those in need.

---

The Daughter, Her Health, Education, and Wedlock. Homely Suggestions for Mothers and Daughters. By William Capp, M. D. Philadelphia and London: F. A. Davis, 1891. Price, $1.00, net.

In this neat duodecimo volume of 144 pages, Dr. Capp has condensed a vast amount of useful information that every woman may read with pro it. It is written in a pleasing style, free from technical language, and clothed in good diction. The importance of knowledge on the subjects of health, education, and wedlock are sufficiently well understood by all persons of intelligence at the present day. It is not competent for our grandmothers to presume, because they were never ill, that their granddaughters may pursue the same lines of life that they followed in the early years of the century. We must deal with the civilization of today in giving advice with reference to health and education to girls of this period, and this without regard to whether their mothers or grandmothers need a doctor or not. In our view, it were infinitely better for the present generation had the grandmothers paid more attention to subjects dealt with in the book before us. We shall be content with saying that it is one of the best of its kind. The publisher presents it in an attractive form.

---

A Compend of Gynecology (Quiz-Compends No. 7). By Henry Morris, M. D., Late Demonstrator of Obstetrics and Diseases of Women and Children in the Jefferson Medical College, Philadelphia. With forty-one illustrations. Philadelphia: P. Blakiston, Son & Co. 1891.

This compend is well prepared by a man who thoroughly understands the details of his subject and who has been sufficiently clear in the text to make the book something more than the ordinary compend. The illustrations are, for the most part, excellent, and will aid in the understanding of the author's plan. Since we must have Quiz-Compends, it is well to have good ones, and Dr. Morris's book is entitled to be classed in that catagory.

---

Diseases of the Digestive Organs in Infancy and Childhood. With chapters on the Investigation of Disease; the Diet and General Management of Children, and Massage in Pediatrics. By Louis Starr, M. D., Late Clinical Professor of Diseases of Children in the Hospital of the University of Pennsylvania, etc. Second edition; illustrated. Philadelphia: P. Blakiston, Son & Co. 1891.

The study of infantile diseases has progressed wonderfully within the last few years. It is no longer competent to trust the dietary management of the infants to the garrulous old aunties and ignorant nurses that formerly assumed the control of this important field. Science and skill are now in vogue where ignorance and carelessness formerly stalked abroad. None of the recent works attracted more attention on the subject under consideration than did Dr. Starr's first edition, which has become exhausted within a few years. This second edition serves to bring the subject forward to the present knowledge of the science, and will be read eagerly by all interested in the management of children. To our mind there is no more important department in the field of medicine, and we trust that every young physician who is training himself in the science of pediatrics will carefully study this admirable treatise.

---

Manual of the Domestic Hygiene of the Child. For the Use of Students, Physicians, Sanitary Officials, Teachers, and Mothers. By Julius Ufflemann, M. D., Professor of Internal Medicine at the University of Rostock. Translated, with the author's kind permission, by Harriot Ransom Wilinowski. Edited by Mary Putnam Jacobi, M. D. Octavo, pp. x. — 229. New York and London: G. P. Putnam's Sons. The Knickerbocker Press. 1891.

A few years ago such a title as this book possesses would have startled the medical world, but now it is a necessity to have a separate treatise on a subject that heretofore only commanded a chapter or two in the general treatise. Dr. Ufflemann has shown his good judgment in permitting his work to be brought out in this country under such favorable auspices. Dr. Putnam Jacobi is one of the most eminent in the department of children's diseases, and has carefully edited the work so as to adapt itself to the American conditions. Every sanitarian, as well as those interested in pediatrics, should carefully study Dr. Uffleman's treatise.

---

Saunders' Pocket Medical Lexicon. Being a dictionary of words and terms used in medicine and surgery. Collected from the highest authorities, and brought up to the present date. By John M. Keating, M. D., (University of Pennsylvania,) and Henry Hamilton, author of A New Translation of Virgil's Æneid into English Rhyme, etc. Philadelphia: W. B. Saunders, 913 Walnut street. 1890.

A pocket dictionary is certainly a great convenience for students, especially when traveling. It seems to us that this is the especial use to which Dr. Keating's compilation will come. It is certainly one of the best of its kind, as the definitions are concise and clear. The type is plain and the press-work excellent. We commend it to those who need such a medical lexicon.

---

Text-book of Materia Medica for Nurses. Compiled by Lavinia L. Dock, graduate of Bellevue Training School for Nurses; Superintendent of Grace Memorial House. New York: G. P. Putnam's Sons. The Knickerbocker Press. 1890.

A new field for bookmaking has been developed since trained or educated nurses have come into vogue. We have had placed before us, in one way or another, almost all sorts and conditions of books in which nurses are interested, but none that have so admirably touched upon materia medica as this one by Miss Dock. It appears, upon careful examination, to be a book that every nurse ought to possess, and to study it well after possessing it. There is scarcely a superfluous word in the book, and its 200 pages are filled with useful knowledge. It is made up in the usual good form in which the publishers present their products.

